# Cytopathological evaluation of pericardial effusions: 192 cases from a tertiary-level healthcare center

**DOI:** 10.55730/1300-0144.5986

**Published:** 2025-01-19

**Authors:** Ayşegül AKSOY ALTINBOĞA, Nur KIVRAK

**Affiliations:** 1 Division of Cytopathology, Department of Pathology, Faculty of Medicine, Ankara Yıldırım Beyazıt University, Ankara, Turkiye; 2 Department of Pathology, Ankara Bilkent City Hospital, Ankara, Turkiye

**Keywords:** Cytology, pericardial effusion, etiology, malignant

## Abstract

**Background/aim:**

There are many different benign and malignant etiologies of pericardial effusions (PEs), which can compress the heart and large vessels emerging from the heart and cause significant clinical findings. The aim of this study was to cytologically examine the underlying diseases causing PEs and to conduct detailed evaluations of underlying cancers in cases of malignant PE, both within the whole study population and according to sex.

**Materials and methods:**

All PE samples obtained between 2019 and 2024 were reevaluated and categorized as nondiagnostic, negative for malignancy (NFM), atypia of undetermined significance (AUS), suspicious for malignancy (SFM), or malignant according to the 2020 International System for Serous Fluid Cytopathology.

**Results:**

A total of 192 PE samples from 184 patients were analyzed, with 137 categorized as NFM (71.4%), 6 as AUS (3.1%), 5 as SFM (2.6%), and 44 as malignant (22.9%). In the NFM group, PE most often developed secondary to cardiac diseases or cardiac operations (61/137, 44.5%). In the malignant group, pulmonary carcinoma metastasis was most common within the whole population (54.5%) followed by breast carcinoma metastasis (15.9%). Lung carcinoma metastasis (69%) was most common among male patients, followed by gastric carcinoma metastasis (23%), and breast carcinoma (38.9%) followed by lung carcinoma metastasis (33.3%) were most common among female patients as the causes of malignant PE. Among patients who were followed for malignancy, malignant PE was found in 64.2% (43/67). In this study, PE had sensitivity of 95.7%, specificity of 100%, positive predictive value of 100%, negative predictive value of 98.5%, and accuracy of 98.9%.

**Conclusion:**

When PE develops in patients being followed for malignancy, there is an extremely high possibility of malignant PE secondary to pericardial metastasis. Cytological evaluation of PE, which has extremely high sensitivity and specificity, is of clinical importance in patient diagnosis and follow-up.

## 1. Introduction

The pericardial fluid (PF), which acts as a lubricant between the pericardium and heart, is 10–50 mL in volume, and when fluid accumulation exceeds that volume it is known as pericardial effusion (PE) [1]. PE is an important clinical finding that can cause compression of the heart and large vessels emerging from the heart, cardiac dysfunction, and cardiac tamponade [2]. Many etiological factors, including infectious agents, chemotherapy (CT), radiotherapy (RT), metabolic problems such as uremia, diseases related to the heart, autoimmune diseases, and malignancies, can cause PE [3]. In the literature, the rate of malignancy varies between 10% and 58% [4–11]. In cancer-related autopsy series, malignant involvement in the pericardium has been reported to range between 2% and 31% [12,13].

Cytological examination is necessary to clarify the cause of PE. In this study, PE samples were reevaluated cytologically and the underlying diseases causing PE were examined. As there is a limited amount of relevant data in the literature, this study aimed to provide detailed evaluations of underlying cancers in cases of malignant PE within both the whole study population and according to sex.

## 2. Materials and methods

This study was approved by the Ethics Committee of Ankara Bilkent City Hospital.

A computerized search was performed for all pericardial cytology specimens obtained at Ankara Bilkent City Hospital between February 2019 and October 2023. The relevant clinical history, demographic data, and radiological and cytopathological results were recorded. Patients with a history of malignancy were initially diagnosed at either our institution or at another institution, as noted in their records.

Samples were centrifuged for 15 min at 1500 rpm. At least 2 slides were prepared for each specimen and a cell block was also prepared when excess PF was available. One slide was stained with May–Grünwald–Giemsa (MGG) and the other was stained with Papanicolaou (PAP) stain. Immunohistochemical staining was performed for cell blocks in selected cases.

The PF cytology cases were originally interpreted and reported by different cytopathologists at the time of initial diagnosis. All slides were independently reevaluated and categorized in this study as nondiagnostic (ND), negative for malignancy (NFM), atypia of undetermined significance (AUS), suspicious for malignancy (SFM), or malignant (M) according to the 2020 International System for Serous Fluid Cytopathology [9] by both authors.

The rate of malignancy (ROM) was calculated and presented as the proportion of cases in each category with serous membrane involvement, which was evaluated as positive when confirmatory test results (histology or cell block section with clear immunohistochemical results) were present or when there was a strong clinical-radiological impression of serous membrane involvement.

The obtained data were analyzed statistically using Microsoft Office Excel 2016 software. Categorical variables were presented using absolute and relative frequencies. Sensitivity, specificity, positive predictive value, negative predictive value, and diagnostic accuracy were calculated.

## 3. Results

A total of 192 PE samples from 184 patients were analyzed, including 98 female and 86 male patients with a mean age of 61.5 years (range: 8–99 years).

Of the 192 samples, 137 (71.4%) were categorized as NFM, 6 (3.1%) as AUS, 5 (2.6%) as SFM, and 44 (22.9%) as M (Table 1). No samples were evaluated as ND. The demographic data and diagnoses of the patients are presented in Table 1.

In the NFM group, PE was determined to have developed most often secondary to cardiac diseases or cardiac surgery (61/137, 44.5%). Less often, the causes of PE were observed to be renal disease (22/137, 16.1%) and CT and/or RT applied to treat a previous malignancy (19/137, 13.9%). The causes of PE among the NFM cases are shown in Table 2.

For the 6 samples in the AUS group, there was a history of known malignancy in only one case. Among the 5 cases with no malignancy, follow-up occurred for cardiac disease in 3 cases, and no underlying factor that could cause effusion could be found in the other 2 cases. Very few cells were observed where no clear differentiation could be made between reactive mesothelial cells and atypical epithelial cells in 5 cases. In one case, a few atypical epithelioid cells with positive Ber-EP4 and MOC-31 in addition to reactive mesothelial proliferation were observed. No malignancy was determined during the follow-up of 5 of the 6 cases. In one patient with a history of breast carcinoma who developed PE, clinical follow-up data obtained after drainage of the effusion were not available. The underlying diseases, cytomorphological appearances, and follow-up results of the AUS group are shown in Table 3.

In 2 of the 5 samples categorized as SFM, atypical lymphoid cells were observed, and as these cells were CD20- and CD79a-positive in the immunohistochemical examination performed with cell blocks, the samples were reported as suspicious for non-Hodgkin lymphoma. In one of these 2 cases, a clinical investigation was conducted for lymphoma but no enlargement of the lymph nodes or solid lesions were identified. In the other case, follow-up data obtained after effusion drainage were not available. Evidently atypical epithelioid cells suspicious for malignancy were observed in the other 3 cases but these cells were very few in number. In 2 of these 3 cases of suspicious epithelial cells, malignant pericardial involvement was observed clinically and radiologically. However, in one of these cases, follow-up data obtained after effusion drainage were not available. The underlying diseases, cytomorphological appearances, and follow-up results of the SFM samples are shown in Table 4.

**Figure 1:**
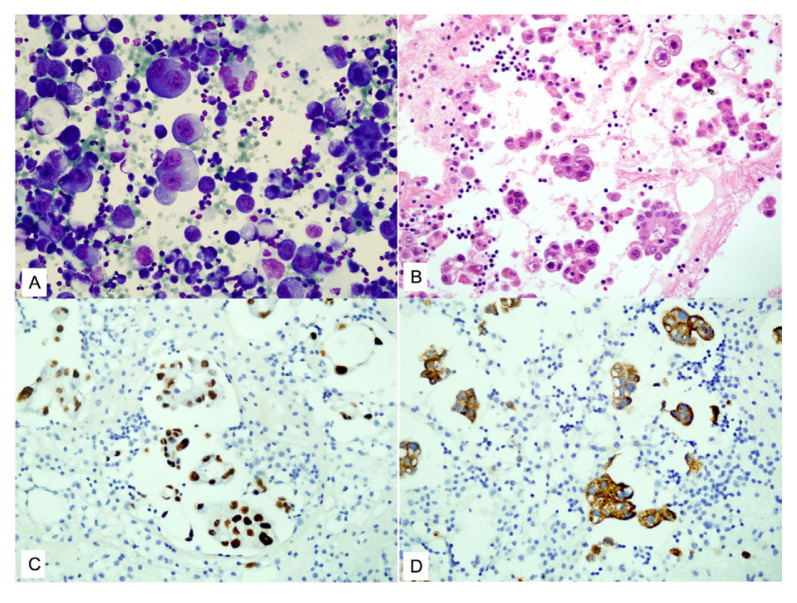
Pulmonary adenocarcinoma metastases: **A)** tumor cells with hyperchromatic enlarged eccentric nuclei and pale vacuolated cytoplasm (MGG, 200×); **B)** cell block section (H&E, 200×); **C, D)** tumor cells showing positive staining for TTF-1 (C) and napsin-A (D) immunohistochemically (200×)

**Figure 2:**
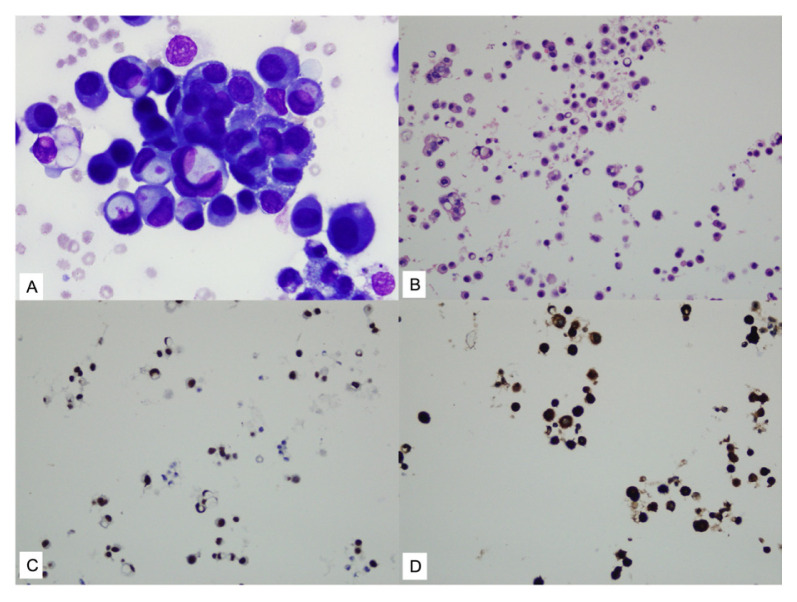
Invasive ductal carcinoma metastases: **A)** tumor cells with hyperchromatic enlarged eccentric nuclei and pale vacuolated cytoplasm, with some tumor cells containing intracytoplasmic musin droplets (MGG, 400×); **B)** cell block section (H&E, 100×); **C, D)** immunohistochemical staining with GATA-3 (C) and mammaglobin (D) showing positive staining in tumor cells (200×).

**Figure 3:**
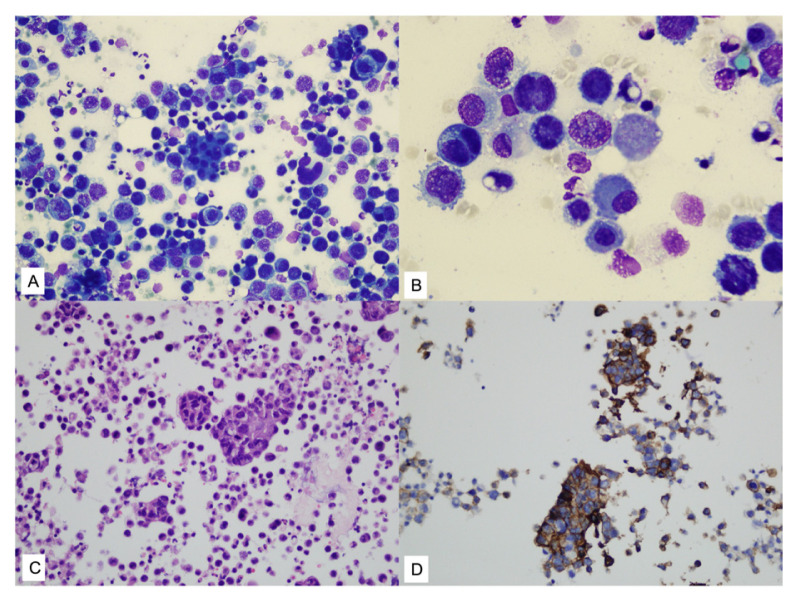
Gastric carcinoma metastases: **A, B)** tumor cells have enlarged hyperchromatic nuclei and increased N/C ratios (MGG: A = 100×, B = 400×); **C)** cell block (H&E 100×); **D)** tumor cells showing positive immunohistochemical staining for Ber-EP4 (100×).

For the 44 samples categorized as M, malignant PF was most often due to lung carcinoma metastasis (n = 24, 54.5%) (Figures 1A–1D). These lung carcinoma cases included 17 cases of adenocarcinoma, 3 cases of squamous cell carcinoma, 3 cases of small-cell lung carcinoma, and 1 case of nonsmall-cell lung carcinoma. Lung adenocarcinoma accounted for 70.1% of the lung carcinoma metastases and 38.6% of all metastases. Following lung carcinoma, the causes of malignant PE were most often breast carcinoma (n = 7, 15.9%) (Figures 2A–2D), gastric carcinoma (n = 6, 13.6%) (Figure 3A–3D), carcinomas of the female genital system (n = 4, 11.4%; 3 ovarian serous carcinomas and 1 cervical squamous cell carcinoma), and prostate adenocarcinoma (n = 1, 2.3%). A diagnosis of malignant mesothelioma was made in one (2.3%) case and in one case of adenocarcinoma the primary site could not be determined.

When malignant PE was evaluated according to sex distribution, lung carcinoma metastasis was determined most often (69%) among male patients, followed by stomach carcinoma metastasis (23%). Among female patients, breast carcinoma metastasis was most common (38.9%), followed by lung carcinoma metastasis (33.3%) (Table 5).

There was a known clinical or radiological tumor diagnosis or the presence of a mass suggesting malignancy in 43 of the 44 cases in the M group. In only one of the cases, the first diagnosis of malignancy was made from the PF. A 61-year-old female patient presented to the Emergency Department with complaints of back pain, shortness of breath, and a history of COVID-19 infection 2 months previously, and in the imaging examination, post-COVID-19 fibrosis in the lungs and PE were determined. A diagnosis of malignant carcinoma metastasis was made in the PE cytological examination. When this patient was investigated further after this result, a mass was found on the right ovary and high-grade serous carcinoma was diagnosed based on the excised mass.

In 67 of the 192 cases, there was a history of malignancy before the development of PE. In 43 of these 67 cases (64.2%), the PE was diagnosed as malignant. The primary tumors in these cases of previously known cancers, the cytological diagnoses of the PE samples, and the malignancy rates are shown in Table 6.

Pericardial excision was performed in only one case in this study, and that sample was evaluated together with the PE sample. This patient was being followed for breast carcinoma and was diagnosed with breast carcinoma metastasis. All other cases were correlated clinically and radiologically.

Recurrent effusion was present in 7 of the 184 patients, including 1 in the M group and 6 in the NFM group. In 6 of these cases 2 effusion samples and in 1 case 3 samples were evaluated. When evaluated again, there was no change in the diagnosis of any case.

Correlation with confirmatory testing was possible in 176 cases (128 NFM, 5 AUS, 3 SFM, and all M). There were no false-positive or false-negative cases. The ROM was 0% for NFM, 0% for AUS, 66.6% for SFM, and 100% for M samples. Sensitivity was 95.7%, specificity was 100%, positive predictive value was 100%, negative predictive value was 98.5%, and diagnostic accuracy was 98.9%.

## 4. Discussion

Pericardiocentesis is the first step for the diagnosis and treatment of PE [1]. However, most PEs are not drained as most cases do not reach a volume that will cause cardiac tamponade. Pleural fluid accumulation is usually seen in patients who develop PE, although variability is seen according to the underlying cause. As there is no risk of cardiac tamponade, pleural fluid drainage as the first step for patients with accompanying pleural fluid is preferred for diagnosis. Therefore, in routine daily practice, cytopathologists encounter fewer PF samples than pleural or peritoneal fluid samples [14]. Correspondingly, there are few publications in the cytology literature that have investigated the reasons for PE occurrence [4–9,15,16]. In studies that have evaluated all types of effusions together, the number of PE cases is generally lower compared to pleural and peritoneal fluid samples [17]. The aim of the current study was to investigate cancers causing malignant PE in detail.

Primary neoplasia of the pericardium is extremely rare [18]. In the presence of malignant PE, the underlying cause is usually a secondary malignancy. The rate of malignant PE was determined to be 22.9% in the present study. There are some studies in the literature that have determined similar rates [5,10,11], but in other studies, this rate has been reported to vary between 10% and 58% [4,6–9]. In autopsy series, pericardial involvement in cancer patients has been reported to range between 15% and 30% [15].

PE in cancer patients can be observed as malignant effusion with metastasis/infiltration of cancer cells into the pericardium. However, benign effusion may develop for reasons such as uremia or thrombocytopenia developing in association with cancer or due to the treatment of malignancy (i.e., secondary to CT and/or RT) [5]. To understand whether the effusion is due to metastases or not and to eliminate the effusion-related symptoms in cancer patients, pericardiocentesis is usually necessary even if pericardial tamponade does not develop [19].

When all the cancer patients of the present study were considered, the rate of malignant PE was 64.2%. In a previous study that evaluated 215 patients with known malignancy who had developed PE, the malignant PE rate was found to be 52.8% [6]. The rate of malignant PE in cancer patients has been reported in the literature to range from 10% to 57% [7–10,20]. In another study that evaluated patients who developed cardiac tamponade while being followed for lung cancer, the malignant PE rate was found to be 73% [21]. Similarly, in the current study, the rate of malignant PE was determined to be 77.4% in patients followed for lung cancer. This rate was found to be 77.8% for breast cancer and 85.7% for stomach cancer. Among cancers represented by extremely small numbers of cases in the present study, this rate was 100% for the 4 cases of cancer of the female genital system and 33.3% for the 3 cases of prostate cancer. Ovarian cancers have been determined to metastasize most often to the liver, pleura, and central nervous system and the probability of malignant PE development has been reported to be extremely low [22]. However, malignant PE was determined in all 3 of the patients with ovarian cancer who developed PE during follow-up in the current study. When one case was further investigated after the diagnosis of malignant PE, ovarian high-grade serous carcinoma was diagnosed. Malignant PE was not determined in patients followed for urothelial carcinoma (n = 3), esophageal carcinoma (n = 1), synovial sarcoma (n = 1), mediastinal germ cell tumor (n = 1), and parotid mucoepidermoid carcinoma (n = 1).

It has been reported in the literature that the cancers causing pericardial metastasis are most often lung cancer, breast cancer, and hematological malignancies [23]. In a study that examined 145 malignant cases of PE, hematopoietic malignancies were reported to be the most frequent cause of malignant PE at the rate of 34.5% [15]. There are different reports in the literature on the topic of cancers causing malignant PE. The most common cause of malignant PE was reported to be breast carcinoma in one study and lung cancer in 4 other studies [4,6,8–10]. In the present study, lung cancer metastasis was the most common cause of malignant PE at the rate of 54.5%, followed by breast cancer metastasis at 15.9% and gastric cancer metastasis at 13.6%.

Among cases of lung cancer, pulmonary adenocarcinoma was diagnosed most often in this study, similar to the findings of Wang et al. [21] and Rodriguez et al. [9]. However, small-cell lung cancer in one study and squamous cell lung carcinoma in another study were determined to be the lung cancers most often responsible for malignant pericardial involvement [5,15].

In the literature, most studies evaluating malignant PE have not evaluated the underlying malignancies according to sex. To the best of our knowledge, there are only two studies that have evaluated malignant cases of PE according to sex [5,7]. In one of those studies, lung cancer was determined to be the most common cause of malignant PE in both male and female patients [5]. In the same study, the second most common cause was metastatic pancreas adenocarcinoma in men and breast carcinoma in women [5]. In the second study, breast and lung cancers were determined at the same rates most often in female patients, whereas in male patients lung cancer was most common as the cause of malignant PE, followed by stomach cancer [7]. In the present study, breast cancer (38.9%) followed by lung cancer (33.3%) were determined as the most frequent reasons for malignant PE in female patients. In male patients, similar to the results for the whole study population, lung cancer (69%) was determined to be the most common cause of malignant PE. This was followed by gastric cancer (23%) in male patients. Some studies in the literature have reported higher rates of malignant PE in women than in men [5,9,24]. In contrast, in the present study, 59% of the malignant cases were diagnosed in male patients. Malignant PE was determined in 30% of all male patients and 18% of female patients.

Dragoescu and Liu determined diffuse large B-cell lymphoma at the rate of 4.3% as a nonepithelial malignant neoplasia causing malignant PE [5]. In the present study, malignant PE was not determined in any of the 5 patients followed for hematopoietic malignancy.

Among cases of malignant PE, secondary malignancy was observed in 97.7% of cases and primary malignancy was observed in 2.3% (n = 1). The patient with mesothelioma causing malignant PE was simultaneously diagnosed with mesothelioma during pleural decortication.

Many etiologies have been reported for benign PE samples, including infections, connective tissue diseases, pericarditis, cardiac surgery, postmyocardial infarction, congestive heart failure, and renal failure [19]. The NFM rate of 71.4% obtained in the present study is similar to previous findings in the literature [5,7,9]. In another study that determined NFM cases at a rate of 74.2%, CT and/or RT treatment for a neoplasm was determined to be the most common etiology of benign PE. In the same study, idiopathic causes, infections, and connective tissue diseases were reported at lower frequencies as causes of benign PE [5]. In the NFM group of the present study, the most common underlying cause was determined to be cardiac disease at a rate of 44.5%, followed by kidney disease and cancer treatments. In another study in which the NFM rate was 84.4%, CT at a rate of 29% was reported to be the most common reason for benign PE, followed by cardiovascular reasons, uremia, and connective tissue diseases at decreasing rates [9]. These differences in benign PE etiologies in the literature can be explained by differences in the patient populations followed by different hospitals.

In PE cytology, cellular component variability can be seen according to the underlying disease. Differentiation of malignant and benign cases can usually be done easily in effusion cytology. A definitive benign or malignant diagnosis was made for the vast majority (94.3%) of the patients in the present study. However, this differentiation can be difficult sometimes, and when there is difficulty in the definitive diagnosis, immunohistochemical examination is helpful in clarifying the diagnosis [9].

When a definitive diagnosis cannot be made by PE cytology, intermediate categories including AUS and SFM are used. Difficulties differentiating between reactive mesothelial cells and malignant epithelial cells and the presence of very few or degenerated evident atypical cells are among the reasons for using these intermediate categories. Overlapping cytomorphological features of reactive mesothelial proliferation and well-differentiated malignant mesothelioma may also lead to the use of intermediate categories. Consistent with findings in the literature, in the present study, cases in these intermediate categories accounted for only 5.7% of the total cases (3.1% AUS and 2.6% SFM) [7,9]. The AUS category included cases for which definitive differentiation between malignant epithelial cells and reactive or degenerated mesothelial cells for small numbers of atypical cells could not be done. Problems of differential diagnosis arising from such cytological features are usually resolved by revealing the nature of the cells immunohistochemically with mesothelial markers such as D2-40 and calretinin [5]. However, the markers that are used to identify carcinoma metastasis, such as Ber-EP4 and MOC-31, stain mesothelial cells at low rates. Therefore, misdiagnosis can be avoided by recognizing the limitations of immunohistochemistry in differential diagnosis [25]. In the immunohistochemical examination performed to differentiate reactive mesothelial cells and atypical epithelioid cells in one of the cases diagnosed as AUS in this study, Ber-EP4 and MOC-31 positivity was seen in calretinin-positive mesothelial cells. However, no tumor was determined in the subsequent clinical follow-up of this case. This highlights the importance of not making a malignant diagnosis based on only Ber-EP4 and MOC-31 positivity.

In this study, very few cells suspicious for malignant epithelial tumors were observed in 3 cases in the SFM category. Malignancies were determined clinically and radiologically in the follow-up of 2 of those 3 cases. In the other SFM cases, the presence of atypical lymphoid cells creating suspicion of non-Hodgkin lymphoma infiltration was observed. In the detailed clinical examination of one of these 2 cases, the possibility of lymphoma was discounted, and in the other case, clinical follow-up information was not available.

No change in cytological category was determined in the reevaluation of 7 cases (3.8%) involving recurrent PE, as has been reported in different studies in the literature [5,7,26]. This demonstrated that a second or subsequent PE cytological examination makes no additional contribution to the determination of malignancy.

This study has shown that when PE develops during the follow-up of patients with malignancy, there is an extremely high possibility of malignant PE secondary to pericardium metastasis. This is more significant in cases of lung cancer in particular, as well as cases of breast, genitourinary system, and gastric carcinomas. Cytological evaluation of PE, which has extremely high sensitivity and specificity, is of clinical importance in patient diagnosis and follow-up. This study has presented cytopathology experiences related to malignancy rates according to sex and the site of the primary tumor in malignant PE cytology.

## Figures and Tables

**Table 1:** Demographic data, diagnostic categories, and malignancy history of the patients.

Diagnostic category	Sex (F/M)	Age, years (range/mean)	Preeffusion malignancy history
NFM (n = 137)	78/59	8–99/62.3	19 cases
AUS (n = 6)	4/2	40–77/60.3	1 case
SFM (n = 5)	3/2	44–89/69.6	4 cases
M (n = 44)	18/26	47–87/58.2	43 cases
Total (n = 192)	98/86	8–99/61.5	67 cases

NFM: Negative for malignancy; AUS: atypia of undetermined significance; SFM: suspicious for malignancy; M: malignant; F: female; M: male.

**Table 2:** Underlying diseases in cases categorized as negative for malignancy (NFM).

Underlying etiologies	Number of cases (%) Total = 137
Cardiac diseases, including myocardial infarction, congestive heart failure, and cardiac surgery	61 (44.5)
Renal diseases, including acute and chronic kidney failure	22 (16.1)
Malignancy-related therapies, including chemotherapy and/or radiotherapy	19 (13.9)
Connective tissue disorders	7 (4 = rheumatoid arthritis, 3 = systemic lupus erythematosus) (5.1)
Hypothyroidism	7 (5.1)
COVID-19 infection	4 (2.9)
Anticoagulant medication	2 (1.5)
Hypereosinophilic syndrome	1 (0.7)
Unknown	14 (10.2)

**Table 3:** Underlying diseases, cytomorphological appearances, and follow-up information of cases categorized as atypia of undetermined significance (AUS).

Underlying etiology	Cytomorphology	Follow-up
Postmyocardial infarction	Few atypical epithelioid cells, reactive mesothelial cells?	No malignancy
Cardiac amyloidosis	Few atypical epithelioid cells, reactive mesothelial cells?	No malignancy
Unknown	Few atypical epithelioid cells, reactive mesothelial cells?	No malignancy
Recurrent pericarditis	Reactive mesothelial proliferation, atypical epithelial cells?	No malignancy
Unknown	Few atypical epithelioid cells, reactive mesothelial cells?	No malignancy
Invasive ductal carcinoma of the breast	Few atypical epithelioid cells, reactive mesothelial cells?	No follow-up

**Table 4:** Underlying diseases, cytomorphological appearances, and follow-up information of cases categorized as suspicious for malignancy (SFM).

Underlying etiology	Cytomorphology	Follow-up
Nonsmall-cell lung carcinoma	Few atypical cells suspicious for carcinoma	Malignant
Prostatic adenocarcinoma	Suspicious for non-Hodgkin lymphoma	No follow-up
Breast carcinoma	Few atypical cells suspicious for carcinoma	No follow-up
Unknown	Suspicious for non-Hodgkin lymphoma	No malignancy
Pulmonary adenocarcinoma	Few atypical cells suspicious for carcinoma	Malignant

**Table 5:** Distribution of the primary focus of malignant PE according to sexes.

Primary site	Male patients, n = 26 (59%)	Female patients, n = 18 (41%)	Total, n = 44 (100%)
Lung	18 (69%)	6 (33.3%)	24 (54.5%)
Adenocarcinoma	12 (46%)	5 (27.8%)	17 (38.6%)
Squamous cell carcinoma	2 (7.7%)	1 (5.5%)	3 (6.8%)
Small-cell carcinoma	3 (11.5%)	0	3 (6.8%)
NSCLC	1 (3.8%)	0	1 (2.3%)
Breast	0	7 (38.9%)	7 (15.9%)
Gastric carcinoma	6 (23%)	0	6 (13.6%)
Genital cancer		4 (9.1%)	4 (9.1%)
Ovarian serous carcinoma	0	3 (16.7%)	3 (6.8%)
Cervix squamous cell carcinoma	0	1 (5.5%)	1 (2.3%)
Prostatic adenocarcinoma	1 (3.8%)	0	1 (2.3%)
Mesothelioma	1 (3.8%)	0	1 (2.3%)
Adenocarcinoma of unknown primary tumor	0	1 (5.5%)	1 (2.3%)

NSCLC: Nonsmall-cell lung carcinoma.

**Table 6:** Cytology results of patients who developed PE during follow-up for malignancy.

Primary malignancy	Malignant	SFM	AUS	NFM	Total	Malignancy rate, %
Lung carcinoma	24	2	-	5	31	77.4
Breast carcinoma	7	1	1	-	9	77.8
Gastric carcinoma	6	-	-	1	7	85.7
Hematolymphoid malignancy*	-	-	-	5	5	0
Female GSC**	4	-	-	-	4	100
Prostate carcinoma	1	-	-	2	3	33.3
Others***	-	-	-	7	7	0
Mesothelioma	1				1	100
Total	43****	3	1	20	67	64.2

* Three cases of non-Hodgkin lymphoma, 1 case of Hodgkin lymphoma, 1 case of acute myeloid leukemia;

** GSC: genital system cancer (including 3 cases of high-grade ovarian serous carcinoma and 1 case of cervical squamous cell carcinoma);

*** 3 urothelial carcinomas, 1 esophageal adenocarcinoma, 1 synovial sarcoma, 1 mediastinal germ cell tumor, 1 parotid mucoepidermoid carcinoma;

**** total of 44 malignant PE diagnoses, as in one case with no previous malignancy information, further investigation determined an ovarian mass and a postoperative diagnosis of high-grade serous carcinoma was made.
